# Males discriminate between substrate-borne cues of conspecific females based on age and mating status in the jumping spider, *Habronattus brunneus*

**DOI:** 10.1098/rsos.240658

**Published:** 2024-10-16

**Authors:** Ellen Humbel, Rebecca Kimball, Lisa A. Taylor

**Affiliations:** ^1^ Department of Biology, University of Florida, Gainesville, FL 32611, USA; ^2^ Entomology and Nematology Department, University of Florida, Gainesville, FL 32611, USA

**Keywords:** sexual selection, chemical ecology, sexual cannibalism, sensory ecology, chemoreception

## Abstract

Sexual selection is often studied with a focus on female mate choice, wherein females evaluate male signals to select an optimal mate. However, in some systems, males should also make careful decisions about the females they choose to court, particularly when faced with the risk of precopulatory sexual cannibalism. Here, we explore the idea that male jumping spiders (*Habronattus brunneus*) may mitigate this risk by responding to female cues probably associated with female aggression and/or receptivity. We tested mature male spiders’ ability to discriminate between substrate-borne cues (i.e. silk and excreta) produced by conspecific females of different ages and mating statuses. We found that males spent more time exploring cues produced by mature, non-mated females compared with either immature females or mated females. Heightened interest in cues produced by females that are sexually mature but not yet mated may allow males to reduce cannibalism risk, reduce wasted courtship effort and increase their reproductive success. The use of chemical and/or tactile cues in jumping spider courtship behaviour has been vastly understudied compared with the ways they use vision; this study provides the groundwork for understanding how these sensory modalities interact.

## 1. Introduction

Historically, sexual selection studies have largely focused on the role of female mate choice as a driver of male courtship behaviour and secondary sexual traits [1]. This concept is primarily supported by the idea that females must endure greater costs of reproduction than males and should, therefore, optimize these interactions by mating with high-quality males [1]. While this logic applies to the courtship dynamics of many animals, there are notable examples in which males also endure great costs in reproduction. In some invertebrate species, such as katydids and bush crickets (family Tettigoniidae), reproduction entails the production of a nutrient-rich spermatophore that is gifted to females [2,3]. In other taxa, males are responsible for brood care, which can involve relatively low-cost egg tending, as in several species of frogs [4,5], fishes [6] and birds [7], or a large investment of time and energy, as in male emperor penguins (*Aptenodytes forsteri*), giant water bugs (*Lethocerus americanus*) and many species of pipefishes and seahorses (family Syngnathidae) [8–10].

Spiders are voracious predators, and in many species, males face the risk of precopulatory sexual cannibalism by females [11]. This cost, like those endured by females, could drive sensory system adaptations that allow males to pursue low-risk courtship interactions. This could be achieved by targeting more receptive or less aggressive females, thereby reducing the risk of being attacked and/or increasing the pay-off if the interaction ends in fertilization. In some spider taxa, factors such as diet and mating status have been shown to influence female aggression toward males, with hungry and previously mated females showing heightened aggression [12–14]. In some cases, males are able to discriminate between females with different reproductive or feeding histories [15–24,25,26]. Males might also be expected to pay attention to female age (i.e. whether a female is sexually mature or not) so as not to waste time and risk cannibalism from an immature female with whom the male has no chance of mating successfully. Given the variation among females that a male may encounter in the field, we might expect males to possess sensory adaptations that allow them to optimize courtship effort by detecting female visual, chemical, tactile or vibrational cues that correlate with female status.

Jumping spiders are understood to rely heavily on vision when foraging and selecting mates [27–32]. However, aspects of female age or experience that might impact aggression may be difficult for males to detect visually. Male jumping spiders have been shown to misdirect courtship toward visually similar heterospecific females in the field [33], which indicates that their ability to quickly identify an ideal mate is imperfect. Given that courting a heterospecific female can have dire consequences in a sexually cannibalistic group of spiders, this courtship risk could drive the evolution of multiple avenues of male perception of female cues. In some spiders, female silk has been shown to contain chemical cues that can vary with the female’s diet, age and mating status [34]—factors that have been shown to influence female aggression and the likelihood of sexual cannibalism [12–14]. Unlike visual cues such as vibrant pigments and structures that are produced once in the development of body scales, chemical cues could provide real-time information about the cue producer. Many pheromones are produced by modifying metabolites from physiological processes [35] and, thus, can provide a snapshot of the physiological state of the individual producing them.

Males that could detect individual female traits linked to aggression or cannibalism (e.g. diet, age or mating status) using substrate-borne cues, such as those left behind by females in dragline silk or excreta, may maximize the likelihood of approaching a viable mate. Because these cues could be chemical or tactile in nature (or even involve multiple sensory modalities), we will use ‘substrate-borne cues’ throughout. Previous studies in jumping spiders have shown that in some species, males use olfactory cues to locate prey and females [25,36] and can distinguish between silk produced by conspecifics and heterospecifics [37,38]. In *Evarcha culicivora*, males and females use chemical cues to determine whether a potential mate has recently eaten a blood-filled mosquito [28,36]. However, little work has been done to understand the nuanced role of silk and/or chemical cues in intraspecific communication in jumping spiders. While the studies cited above provide evidence that some salticid species can use substrate-borne cues to differentiate between species or sex, there has not yet been an effort to test the male ability to differentiate between the species, age and mating status of females in any one species. This approach, which we have adopted here, offers a more comprehensive picture of the sensory repertoire of a single species.

**Figure 1.**
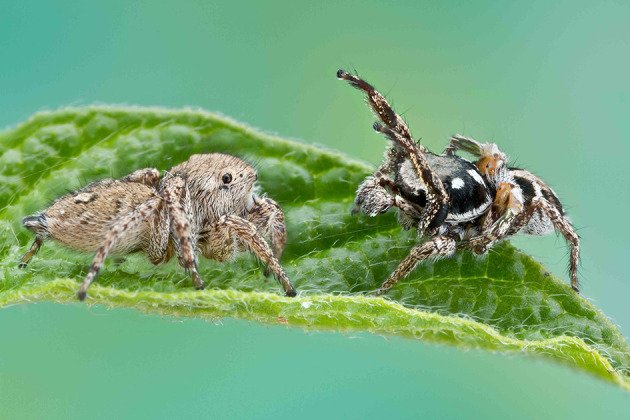
Two *H. brunneus* individuals engaged in courtship. The female (left) faces the male (right) as the male extends its first pair of legs and raises the ornamented portions of its third pair of legs. Thin lines of dragline silk, which may carry chemical cues, can be seen between the spiders and behind the female. Photograph by Colin Hutton.

Little is known about the use of substrate-borne female cues or chemoreception in the genus *Habronattus*, which has been well-studied in terms of male visual cues [31,39,40] and vibratory cues [41,42] during courtship. In *Habronattus pyrrithrix*, males have been shown to spend more time exploring and palpating a filter paper substrate exposed to a conspecific female compared with one that had not been exposed to any female cues [14]. This study expands on this previous work using a congener, *Habronattus brunneus* (figure 1), to better understand male response to substrate-borne female cues that could contain information about female aggression or likelihood of mating.

The goal of our first experiment in this study was simply to establish that *H. brunneus* demonstrates the same ability to differentiate between substrates with and without female cues, as observed in *H. pyrrithrix* [14]. In our second experiment, we tested male responses to substrate-borne cues produced by non-mated conspecific females of different ages (juvenile vs. mature). We predicted that males would prefer substrate-borne cues produced by sexually mature females over those produced by juvenile or penultimate females, as the latter would not confer any reproductive advantage (thus wasting male time and energy while increasing the risk of cannibalism). In our third experiment, we tested male response to female cues produced by sexually mature females that had already mated with another male versus mature females that had never mated. We predicted that males would prefer cues produced by females that had never mated, as females in other *Habronattus* species have been shown to typically mate once and then reject, and sometimes even cannibalize, future suitors (personal observation; see also personal observations by other researchers cited in [43–45]). Natural populations of *Habronattus* can be quite dense, and males are likely to encounter many females (and any cues they produce) while foraging or searching for mates [33]. These experiments allow us to test the ability of male spiders to discriminate among the conspecific female cues that may be most biologically relevant.

## 2. Methods

### 2.1. Spider collection and rearing

We collected spiders in natural areas around Gainesville, FL, from April to July 2019. In this species, juvenile males exhibit a reddish face after their first few moults. Because this trait is not present in juvenile females, we were able to reliably determine the sex of juvenile spiders used in this experiment. All females used in trials were collected as juveniles or subadults to ensure they had not yet mated. We determined the age of female spiders (immature vs. mature) by checking for the sclerotization of the epigynum (reproductive opening of females). After a spider matures (and moults for the final time), this structure is hardened and takes on a dark, shiny appearance. Because males will readily mate multiple times, we did not limit male mating status to non-mated and so collected both immature and mature males for use in trials. We maintained spiders in the laboratory under a 13 : 11 h light : dark photoperiod at approximately 43% relative humidity in individual 5.5 × 5.5 × 12.5 cm acrylic enclosures fitted with artificial plants and small cotton balls moistened with deionized water. Enclosures were separated by opaque barriers to prevent spiders in adjacent enclosures from seeing each other. We administered a standard diet of crickets (*Gryllodes sigillatus*) approximately equal to the spider’s body weight every other day. We misted enclosures with deionized water and replaced the cotton every other day to provide additional humidity (approx. 67% relative humidity within the enclosures immediately after spraying).

### 2.2. Preference experiments

#### 2.2.1. General approach

We conducted three experiments to test male spiders’ ability to distinguish between cues left on filter paper by female spiders. These experiments were identical in design, but each had a different combination of female treatments (detailed below). We conducted all three experiments in the laboratory under the same light and humidity conditions described above. We handled all dishes, surfaces and tools (e.g. blades used to cut filter paper) with gloves and carefully cleaned them with 95% ethanol before and after preparing filter paper or conducting trials to prevent the introduction of aberrant chemical cues. We kept spiders visually isolated from other spiders throughout all stages of the experiments by placing small cardboard barriers (3 cm in height) between dishes. This allowed us to maintain normal lighting conditions while minimizing any visual noise that could affect spiders’ behaviour during trials or filter paper preparation.

**Figure 2.**
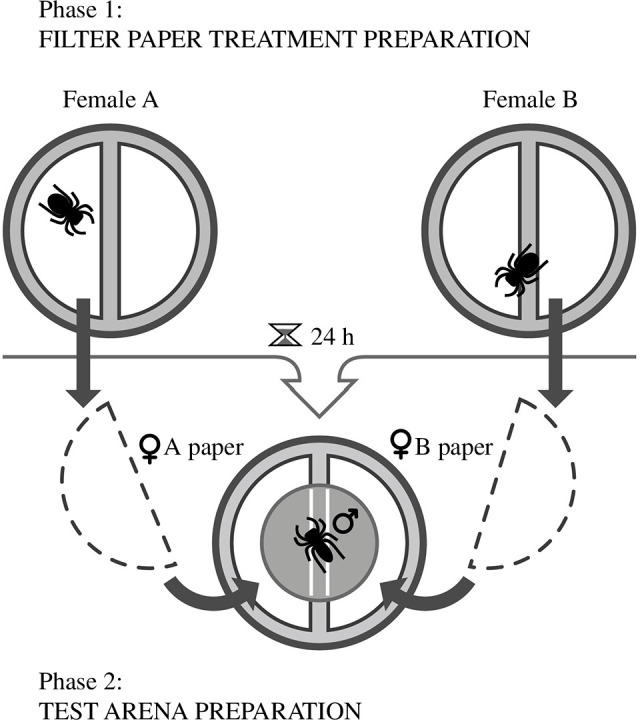
Preparation of filter paper treatments and test arenas. (Top): enclosures used to prepare female-treated filter paper. *Habronattus brunneus* females were placed in a 9 cm petri dish containing two half-pieces of 9 cm filter paper (pre-cut to avoid unnecessary handling during arena set-up) and maintained for 24 h. After 24 h, the spider was removed, and each half-piece of filter paper was transferred to a clean test arena, where it was paired with another half-piece of filter paper. (Bottom): arena used to test *H. brunneus* male response to filter paper treatments in all three experiments. Male is shown in the starting position in a small 3 cm dish within a large 9 cm dish (not shown to scale). Note that in Experiment 1, one of the half-pieces of filter paper was untreated (control) and so did not originate from a petri dish containing a female, as shown here.

We prepared filter paper treatments for experiments by placing female spiders in clean 9 cm glass petri dishes lined with filter paper (figure 2, ‘Phase 1’). To avoid any unnecessary handling of treated filter paper, we cut the clean (9 cm circular) filter paper before placing it in dishes. To do this, we folded each 9 cm piece of filter paper in half and cut along the folded edge, 1−2 mm from the crease. Removing a thin section of paper from the centre (instead of cutting the paper evenly into two 4.5 cm halves) was necessary to create a space between any pair of half-pieces used in the subsequent trials and provided spiders with 2−4 mm of ‘neutral ground’ between the two treatments. This margin acted to create a clear delineation between treatments and prevented transference of chemical cues between the two treatments (figure 2, ‘Phase 2’). We fed female spiders 24−48 h before placing them in the petri dishes lined with filter paper. After each female was allowed to traverse the dish (thus contacting the enclosed filter paper) and produce silk and excreta for 24 h, we removed the female. Immediately after removing the female, we used clean forceps to transfer each half-piece of treated filter paper into a clean 9 cm petri dish, where it was paired with another half-piece of filter paper containing a different treatment (figure 2, ‘Phase 2’). Thus, each female spider provided paper samples for two trials. We randomly determined the side of the dish (left or right) to which each treatment would be placed by flipping a coin and recording the outcome. To ensure truly blind video analyses, this ‘key’ linking the paper treatment to the side of the dish for each trial was entered into a database as metadata and hidden from view thereafter. We repeated this process, pairing filter paper exposed to mature, non-mated females either with a clean half-piece of filter paper that was not exposed to a female (control; Experiment 1) or with paper that had been prepared with complementary female treatments (juvenile females (Experiment 2) or mature, mated females (Experiment 3)). Using this approach, we created pairs of treated paper that males could traverse and inspect during trials (figure 2, ‘Phase 2’). Although each female contributed two samples for use in two separate trials, each trial had a unique combination of female cues. Each male was used in only one trial.

Once dishes were prepared, we introduced males. Each male was transferred from its home enclosure into a 3 cm petri dish and allowed 10 min to acclimatize before we carefully placed the 3 cm dish into the centre of the 9 cm dish (figure 2, ‘Phase 2’). Once the 3 cm dish was placed in the 9 cm dish, we allowed an additional 2 min for acclimatization. To release males, we removed the lid from the 3 cm dish and quickly replaced the lid of the 9 cm dish. The 3 cm dish remaining in the 9 cm dish served as a neutral starting position free of female cues.

#### 2.2.2. Description of treatment groups

*Experiment 1: mature, non-mated female versus control* (*no spider cues present*): females used in this experiment were captured as juveniles or subadults and were maintained in the laboratory until reaching maturity. Females were used in trials 7−21 days after reaching maturity, as *Habronattus* females appear to be most receptive at this age (L Taylor; M Ihle, unpublished data). Each male was presented with one piece of clean, untreated filter paper (control) and one piece of filter paper containing cues from one of the mature, non-mated females described above.

*Experiment 2: age* (*mature, non-mated females vs. juvenile, non-mated females*): mature, non-mated females underwent the same care as those in Experiment 1. We used juveniles that were roughly 50%−75% the size of mature females. We avoided using penultimate spiders (spiders in the stage immediately before their final moult to maturity) because, in some spiders, males show a preference for females that are about to moult into adults. We were more interested in determining whether *H. brunneus* males could distinguish adults from juveniles more broadly.

*Experiment 3: mating status* (*mature, non-mated females vs. mature, mated females*): mature, non-mated females underwent the same preparation as those in Experiments 1 and 2. Females in the ‘mated’ treatment were captured as subadults or juveniles and maintained in the laboratory until reaching maturity. Once these females had been mature for 7−10 days, we paired them with males and monitored them until copulation occurred. Once we had successfully altered the mating status of these females, we kept them under standard laboratory conditions and fed them for another 5−10 days before we used them to prepare filter paper for trials. This 5−10 day period ensured that females could undergo physiological changes associated with gravidity before depositing egg sacs. Any females that deposited egg sacs in this period were excluded from trials, as this event might have affected chemical cues.

### 2.3. Video scoring and statistical analyses

We recorded all trials using Sony Handycam digital cameras mounted above petri dish arenas. As mentioned previously, these videos were scored blind, as metadata linking the paper treatment to the randomly assigned side of the dish was hidden from the analyser. We scored videos by entering the time at which a male spider came into contact with and broke contact with each piece of filter paper. We did not include time spiders spent on the glass lid or sides of the arena or on the holding dish they were introduced from (located in the centre of the testing arena; see figure 2, ‘Phase 2’). We marked each entry ‘left’ or ‘right’ to indicate which piece of paper the spider was touching, then calculated the total time spent on each side (in minutes) over the 30 min trial period. If a spider did not contact both pieces of paper within the first half (15 min) of the trial, that trial was omitted from analyses (Experiment 1: two trials, Experiment 2: four trials and Experiment 3: seven trials), as we cannot draw conclusions about an animal’s preference if it has not experienced the range of options. Only after all video analyses were complete did we employ a query to replace the generic ‘left’ and ‘right’ notations with the treatment conditions contained in our metadata files for each experiment. Our previous study on *H. pyrrithrix* showed that the amount of time males spent on each side of the filter paper was strongly correlated with how many times they palpated the surface of that paper with their pedipalps (a common investigatory behaviour indicative of chemosensation, as the pedipalps are covered in chemosensory hairs) [14,46]. For this reason, we used the time a spider spent on each side of the filter paper as a proxy for male interest. We tested the difference in time an individual male spent on each paper treatment for normality using the Shapiro–Wilk test (R package stats::shapiro.test). We found that the differences were normally distributed in Experiments 1 and 3 but were not normally distributed in Experiment 2. For Experiments 1 and 3, we performed paired *t*-tests in R, using R package rstatix::t_test [47], to compare the total duration in minutes a male spent in contact with each filter paper treatment. Because the Experiment 2 data were not normally distributed, we performed a non-parametric Wilcoxon signed-rank test, using R package stats::wilcox.test, to compare the total duration in minutes a male spent in contact with each filter paper treatment. We calculated effect sizes using Cohen’s *d* tests, using R package rstatix::cohens_d [47].

## 3. Results

**Figure 3.**
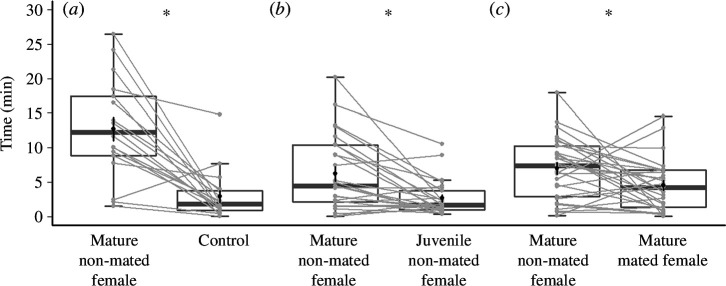
Results of Experiments 1–3 showing male response to filter paper exposed to mature, non-mated females versus (*a*) untreated filter paper (Control), (*b*) juvenile, non-mated females and (*c*) mature, mated females, measured by time males spent on each piece of filter paper. Box plots in dark grey show the median and interquartile range, and the mean ± s.e. is shown in black. Individual males are represented by light grey points with lines indicating the same individual’s time spent on each treatment. Asterisks indicate significant differences between treatment groups. Note that spiders could occupy the lid, rim and neutral starting position during trials, so the total time each individual spent on filter paper is less than the total trial duration.

In Experiment 1, males spent significantly more time on the filter paper that had been exposed to mature, non-mated females (mean (min) ± s.e.; 12.70 ± 1.69) than the untreated paper control (2.94 ± 0.85; *n* = 18; paired *t*‐test; *t*_17_ = −5.29, *p* < 0.0001; Cohen’s *d* = −1.25; figure 3*a*).

In Experiment 2, males spent significantly more time on the filter paper that had been exposed to mature, non-mated females (median = 4.45; interquartile range = 2.20−10.08) than juvenile females (median = 1.72; interquartile range = 1.05−3.50; *n* = 26; Wilcoxon signed-rank test; *V*_25_ = 299, *p* = 0.0010; Cohen’s *d* = 0.73; figure 3*b*).

In Experiment 3, males spent significantly more time on the filter paper that had been exposed to mature, non-mated females (mean ± s.e.; 7.03 ± 0.68) than mature, mated females (4.56 ± 0.83; *n* = 29; paired *t*‐test; *t*_28_ = −2.42, *p* = 0.0223; Cohen’s *d* = 0.45; figure 3*c*).

## 4. Discussion

This series of experiments demonstrates that *H. brunneus* males detect substrate-borne female cues and respond to those cues differently depending on the age and mating status of the female that produced them. Males consistently spent more time exploring filter paper containing cues from mature, non-mated females compared with both juvenile females and mature, mated females. These findings suggest males may use substrate-borne cues to navigate available courtship opportunities.

### 4.1. Use of female cues to improve male success

Males may increase fitness by focusing courtship efforts on females that are more likely to be receptive, as our results suggest. Because females in this genus rarely mate more than once (personal observation; see also personal observations by other researchers cited in [43–45]), *Habronattus* males would probably benefit more from detecting female mating status than those of a polyandrous species where female receptivity does not decline as dramatically after mating. Females that have mated previously could also be more aggressive toward males than non-mated females, as has been shown in other spider taxa [48–50]. Monandry in *Habronattus* could, thus, compound the risk of precopulatory sexual cannibalism as non-mated females become less available over a season. These factors could be powerful drivers of male sensory perception and preferences for cues indicating a female has not mated previously.

Although male spiders may appear uniquely motivated to be discerning when courting, male attentiveness and selectiveness occur across animal taxa [51]. For example, in satin bowerbirds, male success depends in part on their ability to modify their courtship based on female age and condition, as older, more experienced females are less likely to startle during intense male courtship displays. Females signal intolerance of male displays by crouching, and males have been shown to preferentially court females that crouch less [52]. This suggests that by courting more experienced females, males avoid wasting energy on easily startled females that might abruptly fly off during the male’s display. In another example, male guppies (*Poecilia reticulata*) choose to associate more with non-mated females than mated females in both free-swimming scenarios and when given female olfactory cues in the absence of physical and visual contact with females [53].

Given our finding that males spend more time traversing filter paper exposed to mature, non-mated females compared with either juvenile females or mature, mated females, it would be interesting to test male response to other aspects of female variation. Female nutrition has been linked to aggression in several spider species. In the pantropical jumping spider (*Plexippus paykulli*), females show heightened aggression toward courting males when given a low-quality diet [54]. Likewise, in the wolf spider, *Lycosa fasciiventris*, poor female diet and advanced age (in non-mated individuals) increase the likelihood that females will engage in precopulatory sexual cannibalism [13]. The ability to discriminate between chemical cues related to diet could allow males to avoid aggressive females and even increase the likelihood of producing healthy offspring. In the golden-banded orb weaver, *Argiope trifasciata*, male preference for female cues from adjacent versus distant populations has been shown to change depending on female diet [19]. When females were given a natural (varied) diet, males preferred web silk from nearby populations, yet when the diet was standardized to a single prey species, they preferred silk from distant populations [19]. If female diet affects aggression in *Habronattus* as it does in *Plexippus* and non-salticid spider taxa, we would expect males to discriminate between female cues and show preferences for diet treatments that may indicate a female is well-fed (and thus less likely to attack and eat a courting male).

### 4.2. Transmittance and use of substrate-borne female cues

The sensory modality through which substrate-borne female cues are received and processed by *Habronattus* males is still uncertain. Many spiders have been shown to use both airborne (volatile) and tactile chemical cues in communication (reviewed in [34]). Jumping spiders have been shown to use both, but this appears to be largely dependent on species. In a previous study on *H. pyrrithrix*, a closely related congener of *H. brunneus*, males did not demonstrate an ability to differentiate between an airborne cue produced by females and an odourless control presented in an olfactometer [14], though other jumping spiders do use airborne cues [38]. While we assume that *H. brunneus* does not respond to airborne cues, males may use visual cues and chemical/tactile cues in concert, simultaneously perceiving and integrating visual and tactile information. The interplay and relative involvement of these senses in courtship decisions has been touched on in other jumping spider species such as *Lyssomanes viridis* [55] but remains a rich and fascinating field of study.

The specificity of male preference and the ability to detect differences in female age and mating status suggests that there has been some selective pressure driving this sensory response in males. This is also evidenced by the abundance of recorded sexual differences in response to chemical cues in spiders (including jumping spiders), showing that females are much less likely to respond to male cues than vice versa ([34,38], but see *Evarcha* work for an exception [25]). The ability of *H. brunneus* males to distinguish conspecific female cues is interesting, given that males in other closely related *Habronattus* species often seem to court females indiscriminately in the field, including heterospecific females [33]. The observation that males do not appear to be particularly discerning while searching for mates, yet have the capacity to detect differences among conspecific females, suggests that an abundance of sensory information can still lead to imperfect decision-making. It is possible that in the field, males being inundated with sensory information might be less adept at locating and courting conspecific females than they would be in the laboratory (where conditions are optimized for these tasks). Even in this study, where males showed a preference for the ‘optimal’ females (i.e. mature and/or non-mated), they still spent a good deal of time on the ‘suboptimal’ filter paper treatment (i.e. cues from juvenile or mated females). Males also spent time off the filter paper altogether, instead traversing the rim and lids of arenas. It is possible that males could be spending time on ‘neutral ground’ after detecting a female, moving to a higher point in the arena to search for a visual sign of the spider they sensed. When cues from two females were present (Experiments 2 and 3), males may have been more motivated to visually locate females from a higher vantage and away from surfaces contacted by females. This might explain why males spent more total time on filter paper in Experiment 1 (which only included one female treatment) than they did in Experiments 2 and 3. It is important to consider that the ability to detect and process information is complex and prone to error, even in controlled settings.

The differences in male attention to each ‘optimal’ female treatment (i.e. mature and/or non-mated) among these three experiments could be attributed to errors in information processing by males, as suggested above, or could be a product of the unique properties of each type of female cue. For example, it is possible that males showed a stronger preference for mature females over juvenile females because adults produce more silk than juveniles. Jumping spiders tend to deposit dragline silk as they move around, and movement rates in another species of *Habronattus* have been shown to differ between adults and juveniles (L Lietzenmeyer, unpublished data). Future experiments are needed to identify which cues are important for males, and what properties of those cues (e.g. abundance or relative concentration of specific chemical compounds) are responsible for the observed differential response of males.

The results of this study provide the necessary groundwork to further investigate how spiders use chemotactile cues in mate searching, courtship and species recognition, and how chemoreception interacts with other sensory modalities to inform behaviour. Biochemical analysis could help determine what compounds are involved and the physiological origins of those compounds (e.g. dragline silk, nest silk, cuticle, excreta). To date, little is known about sex pheromones in jumping spiders. Chemotactile cues from female spiders of different mating statuses, ages and species could be paired with live females (or video playbacks of females) varying across the same factors to determine how males navigate courtship when both visual and chemotactile cues are present. Such studies could reveal whether different types of sensory information are processed in a hierarchical manner and would provide a fuller understanding of the sensory landscape of jumping spiders.

## Data Availability

The data are available from the Dryad Digital Repository [56].
